# Pdlim7 Regulates Arf6-Dependent Actin Dynamics and Is Required for Platelet-Mediated Thrombosis in Mice

**DOI:** 10.1371/journal.pone.0164042

**Published:** 2016-10-28

**Authors:** Alexander E. Urban, Erin O. Quick, Kaylie P. Miller, Jennifer Krcmery, Hans-Georg Simon

**Affiliations:** Department of Pediatrics, Feinberg School of Medicine, Northwestern University and Stanley Manne Children’s Research Institute, Chicago, Illinois, United States of America; Ludwig-Maximilians-Universitat Munchen, GERMANY

## Abstract

Upon vessel injury, platelets become activated and rapidly reorganize their actin cytoskeleton to adhere to the site of endothelial damage, triggering the formation of a fibrin-rich plug to prevent further blood loss. Inactivation of *Pdlim7* provides the new perspective that regulation of actin cytoskeletal changes in platelets is dependent on the encoded PDZ-LIM protein. Loss-of-function of Pdlim7 triggers hypercoagulopathy and causes significant perinatal lethality in mice. Our in vivo and in vitro studies reveal that Pdlim7 is dynamically distributed along actin fibers, and lack of Pdlim7 leads to a marked inability to rearrange the actin cytoskeleton. Specifically, the absence of Pdlim7 prevents platelets from bundling actin fibers into a concentric ring that defines the round spread shape of activated platelets. Similarly, in mouse embryonic fibroblasts, loss of Pdlim7 abolishes the formation of stress fibers needed to adopt the typical elongated fibroblast shape. In addition to revealing a fundamental cell biological role in actin cytoskeletal organization, we also demonstrate a function of Pdlim7 in regulating the cycling between the GTP/GDP-bound states of Arf6. The small GTPase Arf6 is an essential factor required for actin dynamics, cytoskeletal rearrangements, and platelet activation. Consistent with our findings of significantly elevated initial F-actin ratios and subsequent morphological aberrations, loss of Pdlim7 causes a shift in balance towards an increased Arf6-GTP level in resting platelets. These findings identify a new Pdlim7-Arf6 axis controlling actin dynamics and implicate Pdlim7 as a primary endogenous regulator of platelet-dependent hemostasis.

## Introduction

The primary role of platelets is to seal off an injured blood vessel and prevent further blood loss. Under normal conditions, platelets circulate in the blood as quiescent discs, a form specified by their internal microtubule and actin cytoskeleton. Following vascular injury, platelets rapidly convert to their active forms, which then spread and adhere to the exposed subendothelial collagen at the site of damage [1, 2]. Platelets contain on average seven times more actin by cell volume than other non-muscle cells [1, 3, 4]. This high concentration of actin facilitates the rapid and dramatic actin filament de- and re-polymerization in response to activation [2]. Murine knockout models of several actin-associated proteins, including Talin, Kindlin-3, and WAVE-1 have demonstrated that failure of platelets to spread and adhere leads to thrombocytopenia associated with spontaneous bleeding [5,6,7]. However, much less is known about the role of actin-associated mediators in non-injury thrombosis, another process of great clinical importance.

The PDZ-LIM family of proteins contain multiple binding domains that facilitate dynamic interactions with the actin cytoskeleton, nuclear factors, and signaling molecules, thereby allowing these proteins to regulate diverse biological functions [8]. Most notably, PDZ-LIM proteins have been found to function in cardiac and skeletal muscle development and maintenance [9,10,11,12,13,14,15,16], as well as tumorigenesis [17,18]. However, studies have also suggested a role in platelets [19, 20], and recent work on Pdlim1 (CLP36) revealed that loss-of-function of the protein in mice results in arterial thrombosis [21]. Our group has previously demonstrated that the PDZ-LIM family member Pdlim7 (Lmp4, Enigma) associates with actin [22] and dynamically regulates the subcellular localization and activity of the transcription factor Tbx5 [23,24] during zebrafish heart and fin development [9,10]. Global inactivation of *Pdlim7* in the mouse causes cardiac dysfunction and surprisingly, the loss of Pdlim7 proteins triggers systemic venous and arterial thrombosis, leading to significant postnatal mortality [25]. In the current study, we tested the hypothesis that the enhanced clotting defect is due to platelet dysfunction. We report a new in vivo function for Pdlim7 in regulating platelet hemostasis.

## Materials and Methods

### Animal Care

All protocols involving animals in this work were approved by the Institutional Animal Care and Use Committee of Northwestern University and the Stanley Manne Children’s Research Institute, where mice were housed and bred. All experiments used both male and female C57BL/6 mice, with both WT and *Pdlim7*^*-/-*^ experimental animals generated from *Pdlim7* heterozygous crosses. Lifeact-GFP reporters were generated using the same background strain and breeding performed with double heterozygous crosses to generate WT and *Pdlim7*^*-/-*^ fluorescent mice. Breeding pairs were housed one pair per cage. Mice were given ear tags, genotyped using tissue from an ear punch, and weaned at 4 weeks, at which point they were separated by gender with up to 5 mice per cage. As we have reported earlier [25], about 50% of *Pdlim7*^*-/-*^ mice were stillborn or died within 48 hours of birth without any specific outward indication of poor health that would allow for early euthanasia. The survivors lived over 1 year of age. Animals were cared for by Stanley Manne Children’s Research Institute animal facility staff and monitored daily by laboratory personnel to check the status of pregnant mice, the well-being and growth of pups, and the health of adult mice. Visual examination was used to ensure that mice did not have any health conditions and were not fighting. Retired breeders (after 4 litters) and excess heterozygous mice were euthanized using CO_2_. Cardiac puncture to collect blood was performed using 2–3% inhaled isoflurane to anesthetize mice and cervical dislocation to ensure euthanasia of mice post blood draw. Approximately a total of 500 mice were used for this study.

### Histological analysis

Specimens were fixed in 4% paraformaldehyde, dehydrated, embedded in paraplast, and sectioned on a Leica RM2265 microtome (Leica Microsystems). For embryonic studies, sections were stained with 1% Alcian blue pH2.5/nuclear fast red and Hematoxilin & Eosin, respectively. Sections were imaged under bright-field illumination on a Leica DMR upright microscope equipped with a QImaging Retiga 4000R camera using OpenLab (Improvision) software.

### Blood smear analysis

Bloods smears were performed according to a modified Giemsa staining protocol (Sigma). In brief, a droplet of fresh whole blood was placed on a glass slide and using a second slide spread out in one smooth and quick motion. Slides were fixed in methanol and sequentially stained in May-Grünwald and Giemsa solution, rinsed with water, air dried, mounted and imaged under bright-field illumination.

### Blood collection and platelet preparation

Mouse platelets were obtained following published protocols [26]. Blood was drawn from mice anesthetized with 2–3% inhaled isoflurane by cardiac puncture into 3.2% sodium citrate at a ratio of 1:9 or into heparin for the whole blood smears. To collect platelet-rich plasma (PRP), blood was centrifuged at 86 x g for 8 minutes. To increase platelet yield, the lower phase was washed 3 times with Tyrodes buffer pH7.4 (10 mM HEPES, 12 mM NaHCO3, 138 mM NaCl, 5.5 mM glucose, and 2.9 mM KCl). For washed platelets, the combined PRP was spun at 718 x 28 g for 6 minutes in the presence of prostaglandin I2 (0.1 μg/ml). The platelet pellet was resuspended in Tyrodes buffer pH7.4 (10 mM HEPES, 12 mM NaHCO3, 138 mM NaCl, 5.5 mM glucose, and 2.9 mM KCl) to the appropriate concentration. The platelet resuspension was spun at 510 x g for 30 seconds to pellet contaminating red blood cells. The platelet suspension was removed and platelet counts obtained on a Beckman Coulter AcTdiff2 Analyzer. The platelets were adjusted to concentrations optimized for the assay required and allowed to rest for 45 minutes to 1 hour before using for functional assays.

### Platelet aggregations

Aggregation of washed platelets (225μL) at a concentration of 250,000plt/μL was performed in an aggregometer (Chrono-Log Corp.) with stirring at 37°C. Hepes-Tyrode’s buffer, pH7.4 was placed into a separate cuvette to be used as a blank (set to 100% light transmission). After a stable baseline was established, CaCl_2_ (1mM final concentration) was added, followed by human fibronectin-depleted fibrinogen (0.4mg/mL final concentration; Enzyme Research Laboratories), and finally an agonist of a concentration as described in Results. The extent of aggregation was determined after 10 minutes from the addition of the agonist and expressed as a percent of maximum aggregation. At least n = 5 experiments were performed with blood pooled form 5 mice, in order to have sufficient platelets for each concentration of agonist tested. Collagen-Related Peptide (CRP) was a kind gift from Gilbert White II.

### Platelet flow cytometry

Washed platelets (40μL) at a concentration of 10,000plt/μL in Hepes-Tyrode’s buffer, pH7.4 with CaCl_2_ (1mM final concentration) were incubated at room temperature with various agonists as described in Results. The platelet activation profile was determined with PE- or APC-conjugated antibodies to recognize distinct platelet surface receptors. The reaction was stopped after 10 minutes by adding 5 volumes of 1% BSA in Hepes-Tyrodes buffer, pH7.4 to the samples. Expression of the receptors on the platelet surface was measured by flow cytometry (FACSCalibur) with the following parameters: FSC = E00; SSC = 366; FL2 = 736; FL4 = 770. For each concentration of agonist, at least n = 5 experiments were performed with blood pooled form 5 mice.

### Platelet spreading assay

Washed platelets (5 x 10^7^/ml) were treated with 2 U/mL apyrase and 10 μm indomethacin prior to activation with thrombin (0.5 U/ml). The platelets were then plated on glass coverslips and allowed to spread for up to 45 min at 37°C. Surfaces were washed with Tyrode’s buffer to remove non-adherent platelets, followed by indirect fluorescence immunostaining as described below.

### Mouse embryonic fibroblast (MEF) cell isolation

Primary MEFs were derived from *Pdlim7*^*-/-*^ and WT E13.5 embryos as previously described [27]. The MEFs were cultured in DMEM (Invitrogen) supplemented with 10% heat inactivated fetal bovine serum (FBS), 1% L-glutamine, and penicillin/streptomycin. MEFs were used at low passage numbers (3–5) throughout the studies. Indirect fluorescence immunostaining was performed as described below.

### Immunofluorescence and imaging

Indirect immunofluorescence detection was performed as previously described [23,28]. In short, OCT-embedded tissue was cryosectioned on a Leica CM2050S cryostat (Leica Microsystems). Cryosections, MEFs, or washed platelets were fixed with 4% paraformaldehyde, permeabilized with 0.5% Trition X-100, and blocked with 20% goat serum, 5% 20X blocking solution (50mM NH_4_Cl, 25mM Lysine, and 25mM Glycine), and 0.2% BSA in PBS. Tissues or platelets were incubated with primary antibodies including, anti-Pdlim7 [23], anti-PECAM (BD Biosciences), anti-Versican (Chemicon), anti-Vinculin (Sigma), and anti-α-Actinin (Sigma). Primary antibodies were detected using Alexa 488-, 546-, or 633-conjugated secondary antibodies (Invitrogen). Filamentous actin was detected using Alexa Fluor 488 phalloidin (Invitrogen), and nuclei were stained with DAPI (Sigma). Quantification of the phalloidin signal was done using a Leica DMR upright microscope with epi-fluorescence illumination essentially as described [29,30]. Immunofluorescence and differential interference contrast was visualized on a Zeiss 700 confocal laser scanning microscope with a 100X objective (Zeiss, Inc). Images were processed in Photoshop CS4 (Adobe). Structured illumination microscopy (SIM) super-resolution images were taken on a Nikon N-SIM system. Images were captured using Nikon NIS Elements and reconstructed using slice reconstruction in NIS elements. Post-imaging analysis was performed with NIS Elements (Nikon), Image J (NIH), and MATLAB (MathWorks) software.

### Lifeact-GFP platelet imaging

Male Lifeact-GFP reporter mice [31] were crossed with female *Pdlim7*^*-/-*^ mutant mice to generate LifeactGFP;*Pdlim7*^*-/-*^ compound mice. Live Lifeact-GFP platelets from WT and *Pdlim7*^*-/-*^ genetic backgrounds were prepared as described above (diluted to 2.5 x 10^7^/μl) and imaged using an Nikon N-SIM Structured Illumination Super-Resolution inverted microscope consisting of a Ti-E stand with Perfect Focus, 100 X 1.49 NA TIRF objective lens. Images were taken every 12 s and drift in Z was minimized with Perfect Focus correction. Images were acquired using Nikon NIS Elements and reconstructed using slice reconstruction in NIS elements. Post-imaging analysis was performed using Nikon NIS Elements.

### Western blot and small GTPase pull-down

Washed platelets (2x10^8^/mL) were used either resting or stimulated with thrombin (0.5 U/mL) for 3 minutes. Reactions were stopped by adding ice-cold HEPES-lysis buffer (Pierce Biotechnology) and flash freezing for storage. Fresh or thawed lysates were cleared by centrifugation at 14,000 x g for 10 minutes, 4°C. Protein concentrations in the supernatants were determined by BCA assay according to the manufacturer’s instructions (Pierce Biotechnology) and absorbance measured on a spectrophotometer (NanoDrop 8000, Thermo Scientific). Arf6-GTP pull-downs were performed with 500 μg of the protein lysate in a final volume of 1 mL using an Arf6 Activation Assay Kit according to the manufacturer’s instructions (Cell Bioloabs). Eluents were separated by SDS-PAGE and GTP-bound Arf6 proteins were detected by Western blot. In addition, 20 μg of the lysate was used for SDS-PAGE and Western blot analysis to assess the total amount of Arf6, GAPDH, and Pdlim7 in each sample. Antibodies against Arf6 (Cell Signaling Technology), GAPDH (Cell Signaling Technology), and Pdlim7 [23] were used for immunoblotting. ECL detection (Pierce) was used for visualization and images obtained using a BioRad ChemiDoc MP system. Measurement of relative densitometry was done with Photoshop CS4 (Adobe) and quantification performed with Image J. The ratio of Arf6-GTP in the GST-GGA3 pull-downs to total Arf6 was determined. Each band on the immunoblots was normalized to its paired internal control protein. After normalization, the ratio of each experimental treatment to its paired control treatment was obtained, and the geometric means and 95% confidence intervals were calculated [32]. For resting platelets, independent experiment replicas (n = 7) were performed with blood pooled from 3 mice.

### Statistics

All values are expressed as mean ± SD. Comparisons between two groups were evaluated with the unpaired *t* test. P<0.05 was considered statistically significant.

### Ethics Statement

This study was performed in strict accordance with the recommendations in the Guide for the Care and Use of Laboratory Animals of the National Institutes of Health. The protocol was approved by the Institutional Animal Care and Use Committees at Northwestern University (IACUC animal welfare assurance number A-3995-01; IACUC protocol number 15–019). All surgery was performed under anesthesia, and every effort was made to minimize suffering.

## Results

### Pdlim7 functions in the regulation of platelet-dependent hemostasis

**Fig 1:**
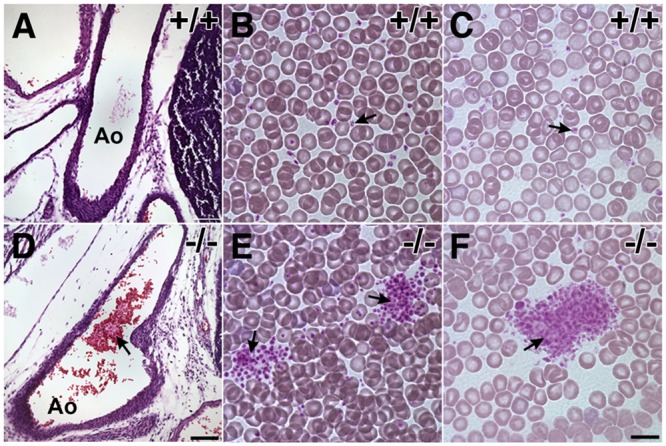
Enhanced platelet aggregation in *Pdlim7*-deficient mice. Pathological analysis of E18.5 embryo histological sections shows clumping of platelets in the aorta of *Pdlim7*^*-/-*^ embryos (D, arrow), but not WT controls (A). Heparinized whole blood smears of *Pdlim7*^*-/-*^ mutant and WT adult mice under bright-field illumination microscopy. Along the smear gradient in areas of higher (B, E) and lower (C, F) blood cell densities small platelets (dark purple) were distributed individually in *Pdlim7*^*+/+*^ mice (B, C), while the platelets in *Pdlim7*^*-/-*^ mice demonstrate aggregation into smaller and larger clumps (E, F). Scale bars = 100 μm (A, D) and 10 μ m (B, C, E, F). Ao = aorta.

In order to study the functional role of Pdlim7 in the mammal, we generated a global knockout mouse. Loss of Pdlim7 predisposes mice to early lethality while survivors develop mild cardiac dysfunction and atrioventricular valve blood clots. Pathological assessment of non-surviving *Pdlim7* KO pups demonstrated systemic occlusive, vascular thrombosis, leading to significant early postnatal lethality [25]. The pre-mortem thrombi detected in the non-surviving *Pdlim7* mutant mice and reduced tail bleed time in adult *Pdlim7*^-/-^ survivors provided a first indication for a functional role of Pdlim7 in regulating hemostasis [25]. For a thrombotic predisposition there are generally three major factors considered: damage to the vessel endothelium, abnormal blood flow, and/or aberrant hemostatic properties of the blood. In previous work we have shown that Pdlim7 localizes to vascular smooth muscle but not endothelium [25], and in this study we ruled out any developmental structural problems in the blood vessels between WT and *Pdlim7*^-/-^ mice (S1 Fig); thus, it is not likely that the excessive clots arise from defects in the endothelial layer following loss of Pdlim7. The decreased bleeding time but normal liver function and plasma coagulation factors in *Pdlim7*^-/-^ mice [25] suggested that the etiology of the hyper-coagulopathy could be platelet-derived, a notion supported by our finding that Pdlim7 is expressed in developing and mature platelets [25]. In this context we note that we did not detect organized thrombi in *Pdlim7*^-/-^ embryos (data not shown); however, we did notice clumping of platelets in *Pdlim7*^-/-^ embryonic vessels, but not in WT controls (Fig 1A and 1D). Additionally, visual examination of heparinized blood smears demonstrated enhanced platelet aggregation in *Pdlim7*^-/-^ adult mice (n = 3; Fig 1E and 1F) compared to WT controls (n = 3; Fig 1B and 1C). Of note, platelet counts were similar between *Pdlim7*^-/-^ mice (n = 7; 518 ± 49 x10^3^ plt/μL) and WT controls (n = 7; 516 ± 62 x10^3^ plt/μL, p = 0.9499). Together, these results indicate a functional role for Pdlim7 in regulating platelet-dependent hemostasis.

### Activation and aggregation of purified platelets appear normal in Pdlim7 mutant mice

**Fig 2:**
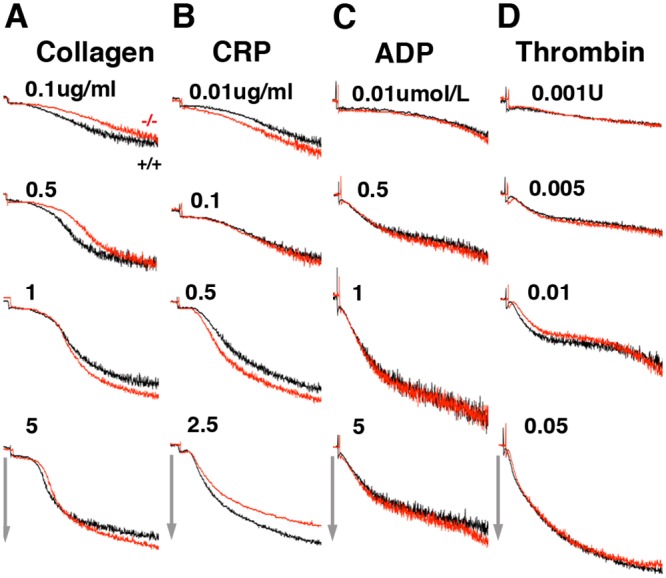
Normal agonist-induced aggregation of isolated *Pdlim7*^*-/-*^ platelets. Washed platelets from WT (black lines) and *Pdlim7*^-/-^ (red lines) mice were stimulated with the following agonists: (A) type I collagen, (B) CRP, (C) ADP, and (D) thrombin. Light transmission was recorded and plotted; arrows indicate 50% light transmission. Graphs represent the average maximum aggregation of Pdlim7^-/-^ platelets relative to normal platelets at a given agonist concentration. Typical aggregation traces of platelets pooled from 5 mice per genotype and representative of n = 6 individual experiments. Abbreviations: CRP: collagen related peptide (AYPGKF).

To characterize the defect in platelets responsible for impaired hemostasis in *Pdlim7* null mice, we used controlled in vitro assays. In order to assess at which step Pdlim7 exerts its function, we first analyzed early stages of platelet aggregation. We compared aggregation of washed *Pdlim7*^*-/-*^ platelets of adult survivors with that of WT platelets in response to several mechanistically different agonists, including G-protein-coupled receptor (GPCR) agonists (ADP and thrombin) and GPVI agonists (type I collagen, collagen-related peptide, convulxin) (Fig 2). The extent of aggregation of *Pdlim7*^-/-^ platelets in response to a range of high and low agonist concentrations was comparable to WT controls, including to the strong platelet activator convulxin (data not shown). We also note that the aggregation curves showed similar peaks for WT and *Pdlim7*^-/-^ mice, corresponding to change in platelet shape after the agonist was added.

**Fig 3:**
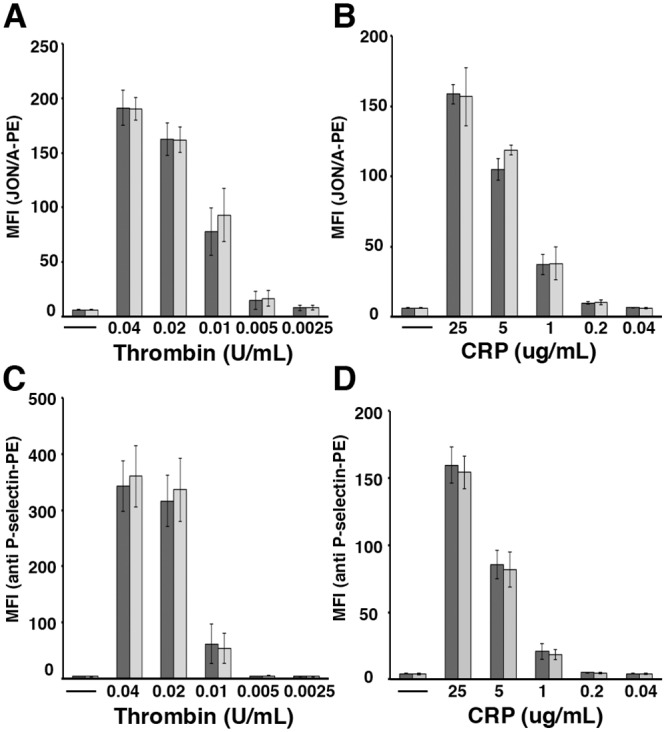
Normal integrin activation and degranulation in isolated *Pdlim7*^*-/-*^ platelets. WT (dark grey bar) and *Pdlim7*^*-/-*^ (light grey bar) washed platelets were stimulated with the agonists thrombin (A, C) and CRP (B, D). Flow cytometric analysis was performed to determine integrin activation (A, B; binding of JON/A-PE) and degranulation (C, D; surface exposure of P-selectin) in response to various agonist concentrations. Results are given as mean fluorescence intensities (MFI) ± SD of 5 mice per genotype and are representative of n = 5 (JON/A-PE) and n = 6 (anti P-selectin-PE), respectively, individual experiments. Abbreviations: CRP: collagen related peptide (AYPGKF).

Second, in parallel with the aggregation studies, we assessed the function of Pdlim7 in platelet activation. Highly diluted washed platelet suspensions were analyzed by flow cytometry for integrin αIIbβ3 activation (JON/A-PE antibody) and α-granule release detected by surface exposure of P-selectin (anti P-selectin-PE) (Fig 3). Activation of *Pdlim7*^*-/-*^ platelets was normal in response to GPCR agonist thrombin as well as to GPVI agonist collagen-related peptide. While we did not observe significant differences upon agonist induced activation between *Pdlim7*^-/-^ and WT mice, we note that these studies as well as the aggregation assays, were performed with washed platelets typically pooled from five mice. Thus, subtle aberrations in individual mice may have gone undetected. These findings suggest that the early stages of platelet activation as measured by agonist-mediated signaling and aggregation responses are not affected in Pdlim7-deficient platelets.

### Pdlim7 localizes to actin filaments in resting and activated platelets in a dynamic spatiotemporal manner

**Fig 4:**
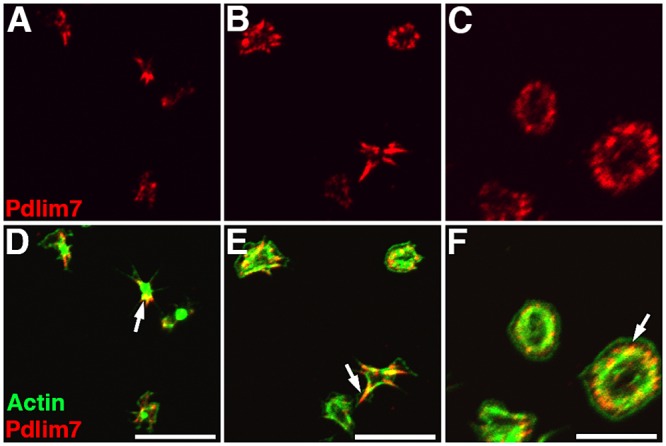
Subcellular localization of Pdlim7 and F-actin during various phases of platelet spreading. Confocal images of thrombin-activated WT platelets spread on glass for 45 minutes, visualizing early (A, D), subsequent (B, E), and late stages (C, F). Double immunofluorescence detection of Pdlim7 and F-actin distribution. Top row (A-C) shows confocal images of WT platelets immunostained for Pdlim7 (red). Bottom row (D-F) shows merged channels of Pdlim7 (red) and F-actin (green). Arrows indicate distribution of Pdlim7 proteins on F-actin filaments at progressively later time points during platelet spreading. Scale bar = 10 μm.

Platelet activation involves a rapid transformation of platelet shape from biconcave discs via filopodia and lamellipodia-extending intermediates to fully spread spherical structures [2]. This alteration of cell morphology is driven by the reorganization of actin, particularly the assembly of new actin filaments [33,34]. Double labeling of Pdlim7 and F-actin in WT platelets spreading on glass revealed co-localization of Pdim7 proteins with filamentous actin (Fig 4). As the actin fibers adopt different morphologies at different stages of spreading, Pdlim7 proteins shift their location along the actin fibers, transitioning from the actin-rich center to the outgrowing actin filaments in filopodia, and finally to a punctate pattern in the concentric actin fiber ring in fully spread cells (Fig 4). In contrast, Pdlim7 was not present in the filamentous actin-free center of the stress fiber ring, the region where the secretory granules are concentrated, nor was it present in lamellipodia or the actin belt at the rim of fully spread platelets (Fig 4).

**Fig 5:**
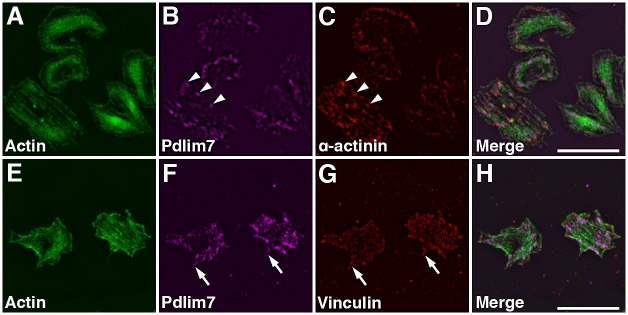
Organization of Pdlim7, actin, and actin-associated proteins during platelet shape change. SIM images of thrombin activated WT platelets displaying intermediate spreading stages. Triple immunofluorescence staining determines the distribution of Pdlim7 in context of the cytoskeletal protein α-actinin and the focal adhesion protein vinculin. Top row shows SIM images of platelets immunostained for (A) F-actin (green), (B) Pdlim7 (magenta), (C) α-actinin (red), and (D) Merge. Bottom row shows SIM images of WT platelets immunostained for (E) F-actin, (F) Pdlim7 (magenta), (G) vinculin (red), and (H) Merge. Arrowheads in panel B and C indicate co-localization of Pdlim7 with α-actinin along actin stress fibers, and arrows in panel F and G point to Pdlim7 distribution in filopodia, just proximal of and abutting vinculin-rich adhesion sites. Scale bar = 5μm.

**Fig 6:**
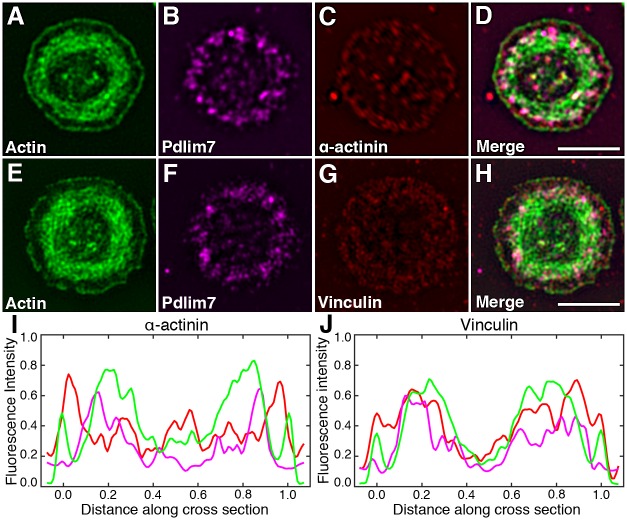
Organization of Pdlim7, actin, and actin-associated proteins in platelets displaying the final round shape morphology. SIM images of thrombin activated WT platelets after completion of spreading. Triple immunofluorescence detection of Pdlim7 distribution in context of α-actinin and vinculin. Top row shows SIM images of platelets immunostained for (A) F-actin (green), (B) Pdlim7 (magenta), (C) α-actinin (red), and (D) Merge. Bottom row shows SIM images of WT platelets immunostained for (E) F-actin, (F) Pdlim7 (magenta), (G) vinculin (red), and (H) Merge. Line scan fluorescence intensity profiles of platelet cross-sections revealing overlapping and distinct localizations of actin (green), Pdlim7 (magenta), α-actinin (red) (I), and of actin (green), Pdlim7 (magenta), vinculin (red) (J). Cell width as well as fluorescence intensities of individual proteins were normalized over seven platelets for each set, respectively. Scale bar = 2.5μm.

Since platelets are significantly smaller than regular cells, we employed structured illumination microscopy (SIM) [35] to characterize the spatial organization of Pdlim7 with respect to other actin-associated proteins, α-actinin and vinculin (Figs 5 and 6). SIM provides a detailed image of protein localization to about ~110nm, which roughly doubles the lateral resolution of conventional light microscopy. In tissue cells, PDZ-LIM proteins, including Pdlim7, are thought to interact with actin fibers via α-actinin [8,36]. In platelets, α-actinin is closely associated with the F-actin network, cross-linking actin filaments and anchoring them beneath the cell membrane and to vinculin-containing adhesion sites [2,35,37,38]. Using SIM the Pdlim7 proteins can be seen to decorate the actin fibers in close association with α-actinin (Fig 5A–5D; arrowheads, Fig 5B and 5C) during the filopodia and lamellipodia extending stages, when F-actin is organized in cable-like structures. At the sites of protruding filopodia, SIM imaging demonstrates extension of Pdlim7 distribution, along with actin, into the filopodia (Fig 5E and 5F). At these intermediate spreading stages vinculin is concentrated at the distal tips of the filopodia, while Pdlim7 remains more proximal and only barely overlaps with the vinculin stain (arrows, Fig 5F and 5G). As the confocal imaging indicated (Fig 4), SIM confirms the complete absence of Pdlim7 from lamellipodia (Fig 5E–5H). This pattern of Pdlim7 association with the actin-binding proteins α-actinin and vinculin changes as the platelets acquire their final morphological stage. In these fully spread platelets α-actinin appears to be predominantly localized to the area right inside the cortical actin belt at the rim of the cell, a location that is void of any Pdlim7 expression (Fig 6A, 6B, 6E and 6F). The F-actin is now organized into the concentric ring and Pdlim7 remains co-localized to this characteristic cytoskeletal structure (Fig 6A, 6B, 6E and 6F). The super-resolution imaging suggests that Pdlim7 is organized along the concentric actin fiber ring in distinct clusters (Fig 6B, 6D, 6F and 6H). SIM shows vinculin to form a diffuse lawn over the platelet surface with a slightly lower abundance in the central secretory granule area. The fluorescent images alone were, however, not conclusive concerning protein co-localizations. We therefore performed line-scans of fluorescence intensity for actin and Pdlim7 in conjunction with α-actinin (Fig 6I and S3 Fig) or vinculin (Fig 6J and S3 Fig) across the platelet diameter, using the cortical actin belt that describes the circumference of the platelet as start and end point. These line scans (mean of seven platelets for each set) demonstrated a protein distribution in concentric ring patterns with various degree of overlap. They confirm that the majority of the Pdlim7 signal is contained within the actin cytoskeletal ring, but also reveal that Pdlim7 is concentrated towards the outer circumference of this actin ring, and no apparent overlap with the α-actinin-rich area just inside the cortical actin belt (Fig 6I). While vinculin appeared fairly evenly distributed in this fully spread platelet stage, the cross-section line scan indicates a higher abundance of fluorescence intensity overlapping with the cortical actin ring and around the outer circumference of the concentric actin fiber ring, which would suggest partial co-localization with Pdlim7 proteins that are concentrated in this area (Fig 6J). The enhanced resolution provided by SIM allowed us to conclude that Pdlim7 has a major role in stabilizing the concentric F-actin ring, but also suggests that it may cross-link adhesion-related proteins to this distinct cytoskeletal structure.

### Loss of Pdlim7 interferes with dynamic actin reorganization in activated platelets

**Fig 7:**
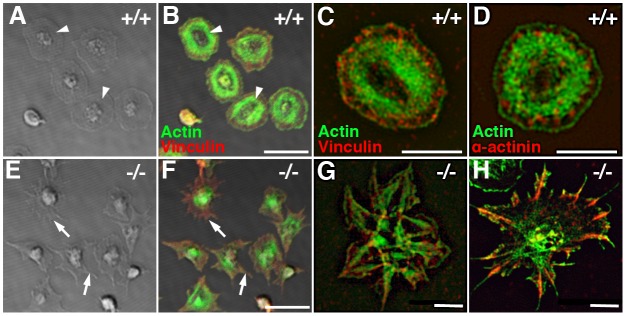
Loss of Pdlim7 disrupts normal actin organization in activated platelets. DIC and confocal images (left hand panel, A, B, E, F) taken of WT and *Pdlim7*^*-/-*^ platelets stimulated with thrombin, spread on glass, and stained for F-actin (green) and vinculin (red). 45 minutes after stimulation, WT platelets were fully spread and displayed an organized F-actin fiber ring (arrowhead, A, B). In contrast, *Pdlim7*^*-/-*^ platelets did not spread, revealed unorganized actin accumulation in the center, and exhibited filopodia-like protrusions with low actin content (arrow, E, F). After completion of spreading, in WT platelets vinculin accumulates along a circle at the microfilament tips, whereas in *Pdlim7*^*-/-*^ platelets it appears to be distributed throughout the platelet body and filling the filopodia-like protrusions (B, F). SIM images (right hand panel, C, D, G, H) show increased detail of actin (green) and vinculin (red) (C, G) as well as actin (green) and α-actinin (red) (D, H) distribution in fully spread WT and Pdlim7^-/-^ platelets. Scale bar (A, B, E, F) = 5μm. Scale bar (C, D, G, H) = 2.5μm.

Given the co-localization of Pdlim7 with actin in normal resting and activated platelets (Figs 4, 5 and 6), we investigated the organization of the actin cytoskeleton in thrombin-activated *Pdlim7*^-/-^ platelets. We performed spreading assays on glass for 45 minutes (n = 4 for WT and Pdlim7^-/-^ platelets), allowing WT platelets to fully spread and assume the peripheral actin stress fiber ring characteristic of this stage as visualized by DIC and confocal microscopy (arrowheads, Fig 7A and 7B). In contrast to the WT, after 45 minutes, the *Pdlim7*^-/-^ platelets displayed a significant accumulation of actin in the center of the platelets, but not the distinct actin stress fiber ring (Fig 7F). Instead, the mutant platelets develop many thin spike-like protrusions that resemble filopodia, but seem to lack the actin density of normal filopodia (arrows, Fig 7E and 7F). While the overall morphological appearance was similar to that of early stage WT platelets, we note that in WT the centrally localized actin is rapidly reorganized, projecting microfilaments outward into filopodia and laterally as they transition to lamellipodia (Fig 4D and 4E). This dynamic morphological change culminating in the round shape characteristic of fully spread platelets is not seen in *Pdlim7*^*-/-*^ platelets. Importantly, the data demonstrate that the absence of Pdlim7 proteins prevents the formation of the concentric actin fiber ring, and super-resolution SIM imaging of activated and comparable staged WT and Pdlim7^-/-^ platelets reveals that the vinculin-rich focal adhesion sites as well as the distribution of the actin filament cross-linking α-actinins are dramatically disorganized in the Pdlim7-deficient platelets (Fig 7C, 7G, 7D and 7H).

Filamentous actin structures in activated platelets were reported to be analogous to those formed in fibroblasts [2,39] and the proteins involved in their formation conserved [40,41,42]. Thus, we employed mouse embryonic fibroblasts (MEFs) isolated from *Pdlim7*^-/-^ and WT mice to investigate whether Pdlim7 would have an influence on cellular architecture and focal adhesions in normal tissue cells. Double labeling of Pdlim7 and vinculin confirmed the absence of Pdlim7 in Pdlim7^-/-^ MEFs and showed that Pdlim7 reaches along the actin filaments into the focal adhesion sites, demonstrating partial overlap with vinculin at the more proximal attachment sites of the elongated WT fibroblasts (S2A Fig). Interestingly, *Pdlim7*^-/-^ MEFs display a very different morphology than WT MEFs; they lose their pointed orientation and adopt a rounder shape (S2B and S2C Fig). In contrast to distally concentrated focal adhesions in WT MEFs, *Pdlim7*^*-/-*^ cells reveal more dispersed vinculin-containing attachment sites within and along the periphery of the cell (S2B Fig). The alterations in cell shape after Pdlim7 elimination were quantified by the ratio of cell length to width, yielding a significant change in circularity (MEFs cultured in low serum, p<0.005; MEFs cultured in high serum, p<0.05; S2C Fig, respectively); thus, demonstrating a problem in cytoskeleton formation and supporting the platelet data. In conclusion, the dynamic distribution of Pdlim7 along actin fibers over time in activated platelets and its co-distribution with α-actinin and vinculin would suggest a role in cytoskeletal remodeling and adhesion.

**Fig 8:**
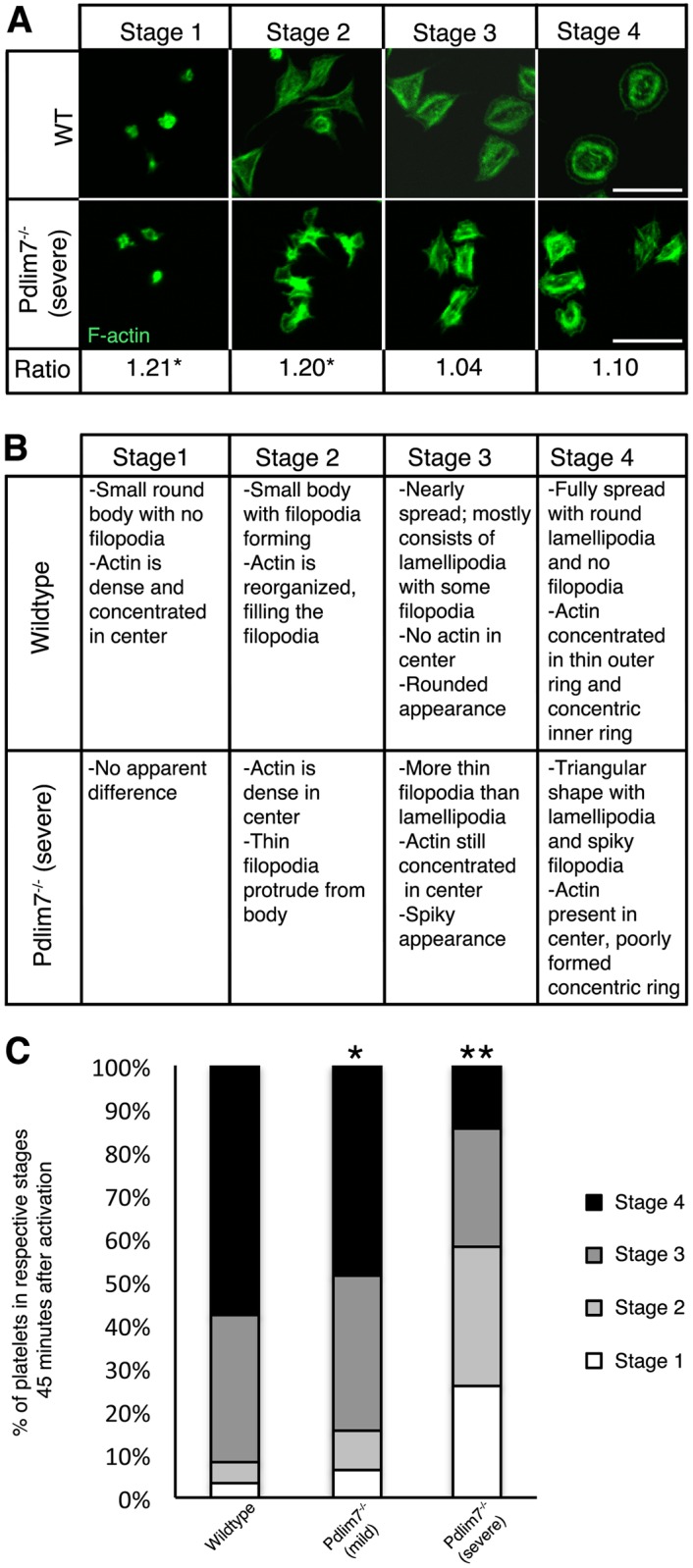
Spreading and F-actin dynamics are altered in activated *Pdlim7*^*-/-*^ platelets. Platelets from WT and *Pdlim7*^*-/-*^ mice were thrombin stimulated and spread on glass for 45 minutes. Fixed platelets were immunostained for F-actin (green), imaged by confocal microscopy, and staged according to the following scheme: stage 1 (resting); stage 2 (filopodia only); stage 3 (filopodia and lamellipodia); and stage 4 (fully spread). (A) Representative images of *Pdlim7*^*-/-*^ platelets presenting a severe phenotype reveal that F-actin remains disorganized and concentrated in the center of the platelets as compared to WT controls. Quantification of the epi-fluorescence intensities of the phalloidin signal of 100 individual platelets in each of the 4 stages and calculation of the ratios of *Pdlim7*^*-/-*^: WT values revealed that the mutant platelets have significantly more polymerized F-actin in the early stages 1 (p<0.05) and 2 (p<0.05), which then normalizes in the later stages 3 (p = 0.59) and 4 (p = 0.19) to values similar to WT platelets. (B) Comparison of platelet shape and F-actin distribution in *Pdlim7*^*-/-*^ contrasted to WT across four stages of spreading. (C) Bar diagram showing the percentage of WT and *Pdlim7*^*-/-*^ morphologies falling into the 4 stage groups. Individual *Pdlim7*^*-/-*^ mice displayed a spectrum of platelet phenotypes. All *Pdlim7*^*-/-*^ platelets have an aberrant actin cytoskeleton with spreading problems–classified as either mild (~90%) or severe (~10%). Both the mild and severe phenotypes exhibit significantly fewer platelets in stage 4 (p<0.05 and p<0.01, respectively) 45 minutes after stimulation as compared to WT. *Pdlim7*^*-/-*^ (severe) platelets have significantly more platelets in stage 1 and 2 (p<0.01) and significantly fewer platelets in stage 3 (p<0.05) and 4 (p<0.01) 45 minutes after stimulation as compared to WT. Scale bar = 5 μm. n = 4 for each genotype and *Pdlim7*^-/-^ phenotype.

In most cases the aberrant early actin organization phenotype observed in the *Pdlim7*^-/-^ platelets was not resolved over time and the platelets remained in a premature deranged stage (Fig 8A). Thus, it appears that the spreading problem is not due to delayed platelet activation but rather, as a consequence of inefficient cytoskeletal remodeling, *Pdlim7*^*-/-*^ platelets cannot exit from the early filopodia protruding stage and become arrested in this intermediary transition morphology.

The characteristic stage-by-stage morphological differences in the Pdlim7-deficient platelets are summarized in Fig 8B. We investigated the actin dynamics further using epi-fluorescence microscopy, as increased actin polymerization will result in increased fluorescence intensity [29,30]. Quantification of the phalloidin signal in *Pdlim7*^-/-^ platelets versus WT controls resulted in increased fluorescence intensity ratios in the null platelets, particularly in the early stage 1 (1.21, p<0.05) and 2 (1.20, p<0.05) images (Fig 8A). These quantitative measurements add to the qualitative imaging data and demonstrate that the Pdlim7^-/-^ platelets are aberrant in early stages of spreading in which actin does not mobilize into normal filopodia. However, we also discovered that *Pdlim7*^-/-^ platelets from individual mice displayed a spectrum of impaired spreading phenotypes as compared to WT controls (Fig 8C). Both, the milder and the severe phenotypes were significantly distinct from WT reaching the final spreading stage (p<0.05 and p<0.01, respectively), with the severe phenotypes detected in approximately 10% of all *Pdlim7*^*-/-*^ mice evaluated.

### Real-time imaging of spreading platelets reveals a temporal and spatial problem in actin cytoskeletal reorganization

The confocal and super-resolution data indicated that loss of Pdlim7 greatly diminishes the capacity of platelets to organize the actin cytoskeleton three-dimensional structure after agonist stimulation. Due to their incredibly small size, live-cell imaging of platelet actin rearrangements is technically challenging. Lifeact-GFP, a 17-amino acid peptide that labels the F-actin cytoskeleton [31], can be used for in vivo monitoring of F-actin structures without interfering with either their formation or function [43]. Lifeact-GFP mice have been also successfully used for functional assays in platelets [44], and we have crossed the *Pdlim7*^-/-^ mice with Lifeact-GFP mice in order to use the actin reporter to visualize the remodeling of actin in *Pdlim7* null platelets in real time.

**Fig 9:**
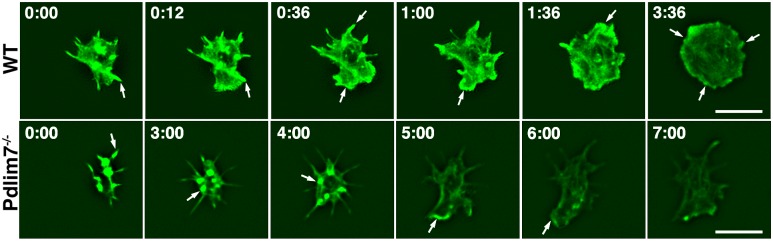
Characterization of actin dynamics by live cell TIRF-SIM imaging. Frames taken from time-lapse TIRF-SIM videos of n = 3 representative Lifeact-GFP mouse platelets from either Pdlim7^-/-^ or WT genetic backgrounds spreading on glass (see S1 and S2 videos). WT platelets (top row) showing the dynamic nature of actin cytoskeleton formation. Pdlim7^-/-^ platelets (bottom row) demonstrating a dramatically altered spatiotemporal-resolved organization of the F-actin, resulting in a significantly deformed cytoskeletal shape. Arrows indicate areas of dynamic polymerization of F-actin filaments. After 3 to 4 minutes of illumination photobleaching becomes apparent; loss of fluorescence intensity was compensated for by adjusting exposure to +3 using Photoshop software for Pdlim7^-/-^ platelets. Time stamp = mins. Scale bar = 5 μm.

Time-lapse TIRF-SIM videos of Lifeact-GFP platelets settling onto glass-bottomed dishes demonstrated that the platelets rapidly adhere and begin to spread (Fig 9, S1 and S2 Videos). As the cells spread and the polymerizing and bundling actin shapes the filopodia, the WT platelets form the characteristic spiky appearance of the early stages of spreading. In subsequent stages, filopodia mature, accumulate more F-actin, and lamellipodia arise from the lateral membrane between filopodia or, alternatively, from the sides of filopodia. Typically after 2 to 3 minutes, the WT platelets reach their final stage, characterized by a fully spread round shape, whose formation is driven by the peripheral F-actin ring structure. In contrast, the time-lapse imaging revealed a different and distinct morphology in the Lifeact-GFP;*Pdlim7*^-/-^ platelets. As the Lifeact-GFP imaging provides a window into the dynamic formation of F-actin fibers, in WT platelets we could observe a wave of F-actin polymerization originating in the center and reaching towards the cell perimeter as the filopodia formed and transitioned to lamellipodia. This wave-like extension of F-actin polymerization did not take place in Pdlim7^-/-^ platelets. Immediately when the Pdlim7-deficient platelets made contact with the glass surface, they displayed a seemingly uncoordinated burst of dynamic extension and retraction of thin, filopodia-like, protrusions. The endogenous actin reporter demonstrated that F-actin is reaching into these spike-like protrusions, which at times were very long. However, filopodia with a more significant F-actin content rarely arose from the surface and formation of proper lamellipodia was similarly scarce. As the images with fixed platelets indicated (Fig 8), significant amounts of actin accumulated in more central locations, often in clusters (Fig 9). Even over an extended time-course of 5 to 7 minutes, the F-actin in the center did not significantly reorganize and, importantly, the real-time imaging did not at any time point indicate remodeling of the F-actin into the characteristic final stage F-actin ring structure. Thus, the Pdlim7-deficient platelets cannot transition into the later spreading stages and they become arrested in a premature filopodia protrusion stage. Along with the co-localization of Pdlim7 and F-actin, the still and real-time imaging data strongly suggest that Pdlim7 regulates actin dynamics and actin cytoskeleton assembly; loss of the protein leads to defects in platelet actin cytoskeleton organization.

### Arf6, a mediator of actin dynamics, is misregulated in Pdlim7^-/-^ platelets

We next sought to identify abnormalities in mediators of actin rearrangements following loss of Pdlim7. The small GTPases of the Ras superfamily have been shown to link signaling events from various platelet receptors to defined outcomes. Particularly, the ADP-ribosylation factor (Arf) 6 is required for activation of the Rho family GTPases and the subsequent cytoskeletal rearrangements needed for full platelet function [45]. Arf6 functions are mediated through its cycling between the GTP/GDP states. The GTP-bound form of the membrane-associated Arf6 is the dominant form in resting platelets and decreases rapidly following platelet activation [45].

**Fig 10:**
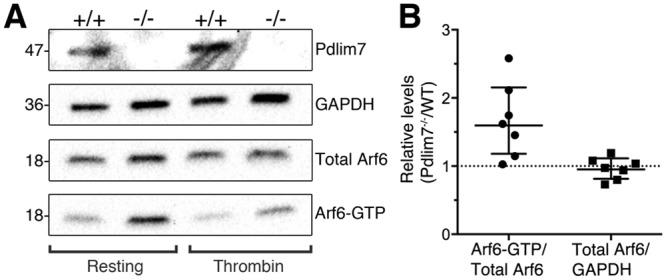
Loss of Pdlim7 results in unbalanced Arf6-GDP / GTP ratios. (A) Western-blot analysis using anti-Pdlim7 antibodies of washed platelets from WT and *Pdlim7*^*-/-*^ mice demonstrates presence of Pdlim7 proteins in WT, but not *Pdlim7*^*-/-*^ platelet lysates (Pdlim7). Anti-Arf6 antibodies detected similar amounts of total Arf6 in *Pdlim7* null platelets (Total Arf6), normalized to the internal GAPDH control (GAPDH). The protein lysate clarified supernatants were used to pull down Arf6-GTP, followed by Western bot detection using anti-Arf6 antibodies (Arf6-GTP). Comparison of resting WT to resting *Pdlim7*^*-/-*^ platelets revealed increased Arf6-GTP to total Arf6 protein ratios following loss of Pdlim7 (A, left hand panel). (B) Graphs show individual data points normalized to control along with geometric mean and 95% confidence interval (5% CI). 95% CIs that do not cross the dotted line at y = 1 represent significant differences relative to the control at p = 0.05, n = 7. Thrombin treated platelets showed a reduced level of Arf6-GTP as compared to resting platelets as would be expected after agonist stimulation (A, right hand panel). The Western blots are representative of n = 7 (resting) and n = 2 (thrombin stimulated) independent experiments with platelets pooled from 3 mice, each experiment.

Since the GTP/GDP cycle of Arf6 is linked to platelet activation and actin dynamics, we performed Western blots for total Arf6 and Arf6-GTP protein levels in non-stimulated washed platelets from *Pdlim7*^-/-^ and WT mice. We detected an altered ratio of Arf6-GTP to total Arf6 protein levels in platelets following loss of Pdlim7 (n = 7; Fig 10A and S4 Fig). In repeated experiments we found the total Arf6 levels between *Pdlim7*^*-/-*^ and WT platelets similar, as controlled to internal GAPDH proteins. In contrast, the Arf6-GTP pull-down assays clearly demonstrated a significantly enhanced level of Arf6-GTP to total Arf6 protein in the resting *Pdlim7*^-/-^ platelets as compared to WT (p<0.05; geometric mean of ratios: 1.572 95% confidence interval lower bound 1.086 upper bound 2.277 at n = 7; Fig 10B). In thrombin-stimulated platelet extracts, the Arf6-GTP level is expected to rapidly decrease [45]. Accordingly, after three-minute thrombin activation of washed platelets, we detected a drop in Arf6-GTP signal intensity on Western blot in both, WT and *Pdlim7*^-/-^ platelet protein extracts (Fig 10A). These molecular data provide the first evidence that Pdlim7 modulates cell shape changes associated with platelet activation likely via its regulation of Arf6.

## Discussion

Genetic inactivation of Pdlim7 in the murine model causes spontaneous systemic thrombosis, leading to early perinatal lethality. In the current study, we focused our analyses into the etiology of the prothrombotic phenotype on the *Pdlim7*^*-/-*^ mutant survivors. Despite approximately half of *Pdlim7*^-/-^ mice escaping premature death, the data presented reveal that the survivors exhibit hemostatic dysfunction, but also display significant animal-to-animal variation. In addition, the phenotypes of the survivors are expected to be milder, which may preclude an appreciation of the full underpinning problems caused by the absence of Pdlim7 proteins.

### Pdlim7 is needed for actin cytoskeletal organization and completion of platelet shape change

Like other PDZ-LIM family members, Pdlim7 is an actin-binding protein [8]. In previous work we have shown that Pdlim7 binds to polymerized F-actin, but not to latrunculin A or cytochalasin B destabilized filamentous actin [23]. In addition, in transfected COS-7 cells we found the relative overexpression of Pdlim7 causes significant actin stress fiber formation [22,23]. In contrast, MEFs generated from *Pdlim7*^*-/-*^ embryos demonstrate cytoskeletal problems resulting in a deficiency in forming a cellular axis, in addition to enhanced number and mislocalized focal adhesion sites (S2 Fig). This phenotype is impressively confirmed in platelets, where Pdlim7 is dynamically distributed along actin fibers in normal spreading platelets (Fig 4). Therefore, our observation in *Pdlim7*^-/-^ platelets that the peripheral F-actin fiber ring is not established (Figs 7 and 8) may be a function of a missing filament-organizing feature of Pdlim7 proteins. Thus, the experimental evidence would suggest that Pdlim7 has a role in crosslinking actin filaments to networks or bundling of actin filaments; loss of Pdlim7 may directly or indirectly via lack of binding to α-actinin, interfere with actin cytoskeleton organization into higher order structures, leading to aberrant platelet morphologies.

This actin-organizing role appears to be an emerging theme of PDZ-LIM proteins as in other cells, Pdlim4 (RIL), Pdlim2 (Mystique), and Pdlim1 (CLP36) also localize to and influence actin stress fiber organization [20,46,47,48,49]. For example, overexpression of Pdlim4 in osteosarcoma cells causes continuous assembly and collapse of stress fibers, resulting in rapid changes in cell shape [49], while knockdown of Pdlim1 in BeWo cells leads to loss of stress fibers and focal adhesions [47]. Our in vitro MEF and platelet studies provide the first insights into a fundamental cellular function for Pdlim7 in actin-associated processes, particularly in cytoskeletal organization and rearrangement. However, the differential localization of Pdlim7 in resting and activated platelets seems paradoxical: under resting conditions Pdlim7 apparently stabilizes actin filaments, preventing cytoskeletal changes; conversely, in activated platelets Pdlim7 promotes actin reorganization. Because of their small size, it is difficult to resolve these potentially disparate functions directly in platelets. Fibroblasts are useful models to functionally dissect actin cytoskeletal reorganization, and future cell biology studies with *Pdlim7*^*-/-*^ MEFs will aim to address these mechanistic questions in more detail.

### Pdlim7 functions in regulating actin dynamics and filament network formation

Remodeling the actin cytoskeleton following platelet activation proceeds in a sequence of morphological steps that involve fragmentation of existing filaments, formation of new filaments, crosslinking actin filaments into networks to form intermediate spreading stages, and bundling filaments to protrude filopodia [1,3]. The actin assembly occurs at the cytoplasm-membrane interface and is mediated by proteins that regulate actin dynamics as well as proteins that bind directly to actin and modulate actin structures [1,33,34].

The Ras-related small GTPases of the Arf family play critical roles in hemostasis. Arf6 is a membrane-associated family member, and upon platelet activation it shifts from the active GTP-bound to the GDP-bound state, which results in the activation of Rho GTPases, RhoA, Rac1, and Cdc42 [45], Cdc42/Rac1-dependent activation of PAK [50], Pak1/ROCK activation of LIM-kinase 1 [51,52], and subsequent regulation of cofilin-controlled actin polymerization/ depolymerization leading to platelet shape changes [19].

Arf6 functions are not mediated just by the GTP-bound state, but through its cycling between states [53,54]. Similarly, the downstream regulation of actin cytoskeletal dynamics is mediated by cycling of cofilin between its dephosphorylated form that binds actin and its phosphorylated form that prevents actin binding [55,56]. During platelet activation and actin reorganization, counteracting cofilin phosphatase and LIMK-1 pathways regulate cofilin phosphorylation [19]. Binding of cofilin to actin filaments promotes depolymerization. However, cofilin-induced filament severing also results in the generation of free barbed ends, which serve as a substrate for accelerated actin polymerization. Thus, cofilin supports depolymerization and polymerization of actin, depending on the cellular content of actin filaments relative to actin monomers and free barbed ends [57].

In this context, we note that while *LIMK1*^*-/-*^ platelets showed reduced actin polymerization only late after stimulation [58], the specific inhibition of the Rho kinase that phosphorylates and activates LIMK1 results in significant F-actin level increase in unstimulated platelets [19]. This finding is reminiscent of the increased F-actin ratios in Pdlim7-deficient platelets (Fig 8). Thus, the increased Arf6-GTP level in *Pdlim7*^*-/-*^ platelets may indeed result in the increased F-actin content observed in resting platelets, indicating a defect in regulating actin dynamics. The altered balance of the GTP-bound state of Arf6 in *Pdlim7*^*-/-*^ platelets provides the first mechanistic insight into the function of Pdlim7 as a regulator of actin dynamics driving shape change in activated platelets. As Arf6 is involved in protein trafficking, in future experiments employing MEFs it would be of interest to explore whether Pdlim7’s function could be related to the activation of Arf6 and Arf6’s subsequent trafficking of Pdlim7 to actin filaments in order to stabilize them.

### A new Pdlim7-Arf6 axis controlling actin dynamics

Our findings support a model in which Pdlim7 functions in the formation and organization of actin fibers, in particular the peripheral actin ring of fully spread platelets, which is critical for correct cell shape and later for clot retraction. Our blood smear assay (Fig 1) as well as the excessive filopodia-like protrusion phenotype, including the mislocalization of vinculin-containing attachment sites (Fig 7) suggest that the misshapen Pdlim7^-/-^ platelets are more adherent than their normal counterparts. In addition, as Pdlim7^-/-^ platelets cannot properly exit the transition morphology (Figs 8 and 9), they consequently remain in an intermediate activation step longer, resulting in the overall activation of more platelets. Both scenarios would likely result in the accumulation of platelets to form larger clots.

It is possible that compensatory mechanisms by other PDZ-LIM proteins may rescue the lack of Pdlim7 proteins, although at a slower pace or by a slightly different mechanism. In this context, it is interesting that Pdlim7 (Figs 4, 5 and 6) and Pdlim1 [21] both localize to actin stress fibers in platelets. Moreover, both proteins, Pdlim1 or Pdlim7, appear to be key regulators of thrombosis, although the underpinning mechanisms are clearly distinct and may be due to the different protein structure and/or subtle differences in spatiotemporal localization on the actin cytoskeleton [21]. *Pdlim1*^*-/-*^ platelets display hyperactivity of glycoprotein VI (GPVI) signaling, a pathway that appeared normal in *Pdlim7*^*-/-*^ platelets (Figs 2 and 3). However, our current work demonstrates that Pdlim7 operates upstream of Arf6 signaling and affects actin dynamics as well as spatial and temporal platelet shape changes (Figs 8, 9 and 10). It is of interest that a recent report described Arf6 knockout platelets to exhibit an enhanced spreading phenotype [59]. This is the inverse phenotype displayed by Pdlim7 knockout platelets and would support the proposed Pdlim7-mediated regulation of Arf6 activity. An alternative consideration would be that loss of Pdlim7 could cause enhanced stabilization of the actin filaments in the platelet disc and early filopodia-forming stages. Thus, it takes longer for the platelets to reorganize their actin into stress fibers despite potential compensation by Pdlim1. Of note, during platelet spreading, Pdlim1 is highly concentrated along the radially outgrowing actin filaments, but is absent from the F-actin-rich center [20], whereas, at this stage, Pdlim7 is concentrated in the F-actin-rich center and absent from the outgrowing filaments. These data suggest that Pdlim1 would have the ability to compensate for Pdlim7 at later stages of platelet spreading, but not at the earlier stages. The generation of compound knockout mice will clarify this point in the future.

Nothing is currently known about Pdlim7 mutations in humans; however, considering these new findings, future studies investigating the clinical relevance of Pdlim7 would be of great interest. In the murine model, a deficiency in Pdlim7 causes spontaneous systemic venous and arterial thrombosis, resulting in significant lethality. Our data point to a new function for the actin-associated Pdlim7 protein in regulating platelet activation and shape change through the Arf6 signaling pathway. The Pdlim7 knockout mouse may serve as a unique model to better understand how platelet activation and actin remodeling is coordinated at the molecular level. This is the first demonstration that a structural problem in the cytoskeleton can transform platelets in a hyperactive state, which will likely aid in our understanding of human coagulopathies and may provide a novel target for the next generation of antithrombotic therapy.

## Supporting Information

S1 Fig Filamentous actin organization in the vasculature smooth muscle of *Pdlim7*^*-/-*^embryos appears normal. Immunohistochemistry of sagital sections through the aorta of an E15.5 WT (A, C) and *Pdlim7*^*-/-*^ embryo (B, D) stained for actin (green), versican or PECAM (red), and DAPI nuclei (blue). Scale bar = 20 μm. Ao = aorta.
(TIF)

S2 Fig Altered cell shape and vinculin distribution in *Pdlim7*^*-/-*^ MEF cells. MEFs were isolated from *Pdlim7*^*-/-*^ and WT mice and cultured in MEF medium plus 0.1% or 10% FBS. Cells stained with antibodies specific for Pdlim7 (red) and vinculin (green); control DAPI nuclei (blue). In contrast to distally concentrated focal adhesions in WT MEFs (A), *Pdlim7*^*-/-*^ cells display many focal complexes disorganized within and along the cell periphery (B). The shapes of primary *Pdlim7*^*-/-*^ MEFs and WT controls were further analyzed for circularity and the difference of the morphometric parameters quantified (C).
(TIF)

S3 Fig Intensity of protein fluorescence signals across the width of fully spread platelets. Line scan fluorescence intensity profiles of individual platelet cross-sections displaying distribution of actin (green), Pdlim7 (magenta) in conjunction with α-actinin (red) (left panels), and vinculin (red) (right panels). Intensities were analyzed on a line segment (width 380 nm) passing close to the center of each cell. Length of the line scan was normalized to 0 to 1 distance (x-axis) (left panel 3.47–6.04 μm; right panel 3.22–5.66 μm, n = 7 platelets, each set), using the cortical actin ring as start and end point. Along the diameter of the cell, fluorescence intensities were quantified and normalized to 0 to 1 (y-axis), representing minimum and maximum fluorescence of the individual proteins for each platelet scanned. The individual scans of a given protein were averaged over 7 platelets for each set (bold line). The mean normalized fluorescence profiles of each protein set were used for line scan panels of Fig 6I and 6J, respectively.
(TIF)

S4 Fig Original Images of Western blots. Top image shows Western blot processed with primary antibodies against Arf6 and GAPDH, and secondary goat anti-rabbit HRP-coupled antibody for visualization of proteins. Bottom image shows the same membrane stripped and re-probed with primary antibody against Pdlim7 in combination with goat anti-rabbit HRP-coupled secondary antibody as before. Protein lysates from WT and Pdlim7^-/-^ mice were analyzed, in addition to samples from pull-downs that display the active, GTP-bound form of Arf6. Labels along the top of the images indicate sample loading. Images were obtained using a BioRad ChemiDoc MP system.
(TIF)

S1 Video Real-time imaging of actin distribution in Pdlim7^-/-^ platelets. Representative time-lapse videos of spreading platelets from LiveactGFP (5/11/16 sample) and LifeactGFP; *Pdlim7*^*-/-*^ (5/19/16 sample) mice revealing a spatiotemporal defect reorganizing the actin cytoskeleton in Pdlim7-deficient platelets.
(MOV)

S2 Video Real-time imaging of actin distribution in Pdlim7^-/-^ platelets. Representative time-lapse videos of spreading platelets from LiveactGFP (5/12/16 sample) and LifeactGFP; *Pdlim7*^*-/-*^ (5/24/16 sample) mice revealing a spatiotemporal defect reorganizing the actin cytoskeleton in Pdlim7-deficient platelets.
(MOV)
