# Supplementary figures and images for: Pdlim7 Regulates Arf6-Dependent Actin Dynamics and Is Required for Platelet-Mediated Thrombosis in Mice
Source: PLoS One. 2016 Oct 28;11(10):e0164042. doi: 10.1371/journal.pone.0164042 (PMC5085081; doi:10.1371/journal.pone.0164042)

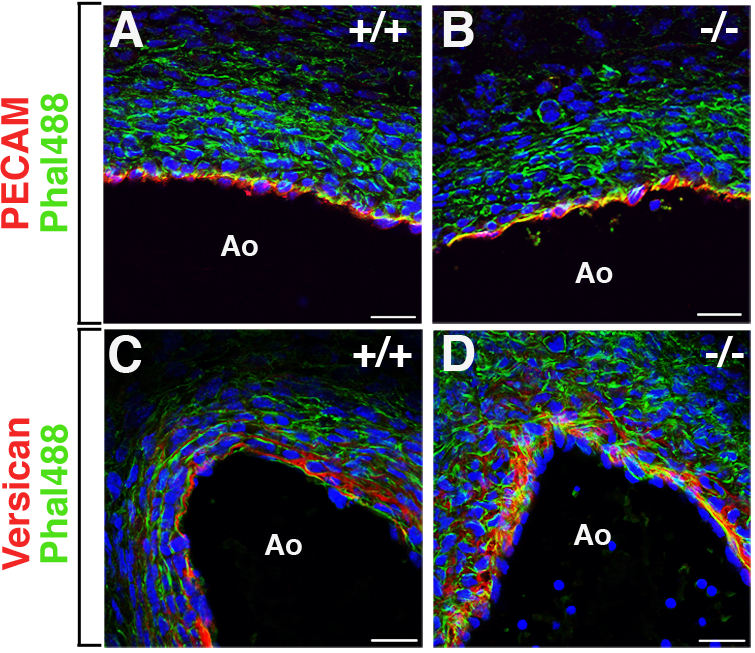

Supplement: S1 Fig — Immunohistochemistry of sagital sections through the aorta of an E15.5 WT (A, C) and Pdlim7-/- embryo (B, D) stained for actin (green), versican or PECAM (red), and DAPI nuclei (blue). Scale bar = 20 μm. Ao = aorta. (TIF) [file pone.0164042.s001.tif]

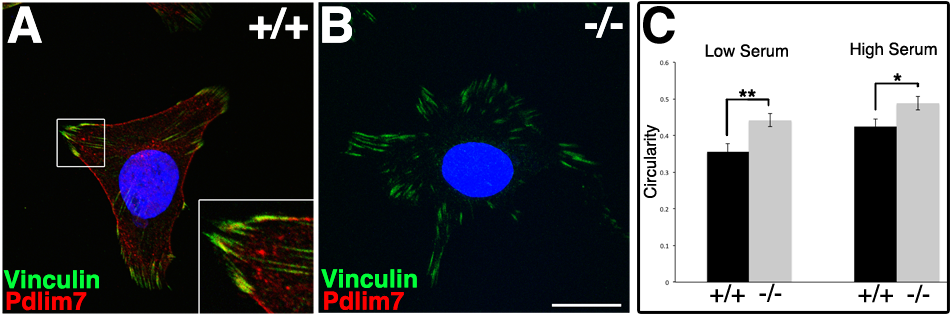

Supplement: S2 Fig — MEFs were isolated from Pdlim7-/- and WT mice and cultured in MEF medium plus 0.1% or 10% FBS. Cells stained with antibodies specific for Pdlim7 (red) and vinculin (green); control DAPI nuclei (blue). In contrast to distally concentrated focal adhesions in WT MEFs (A), Pdlim7-/- cells display many focal complexes disorganized within and along the cell periphery (B). The shapes of primary Pdlim7-/- MEFs and WT controls were further analyzed for circularity and the difference of the morphometric parameters quantified (C). (TIF) [file pone.0164042.s002.tif]

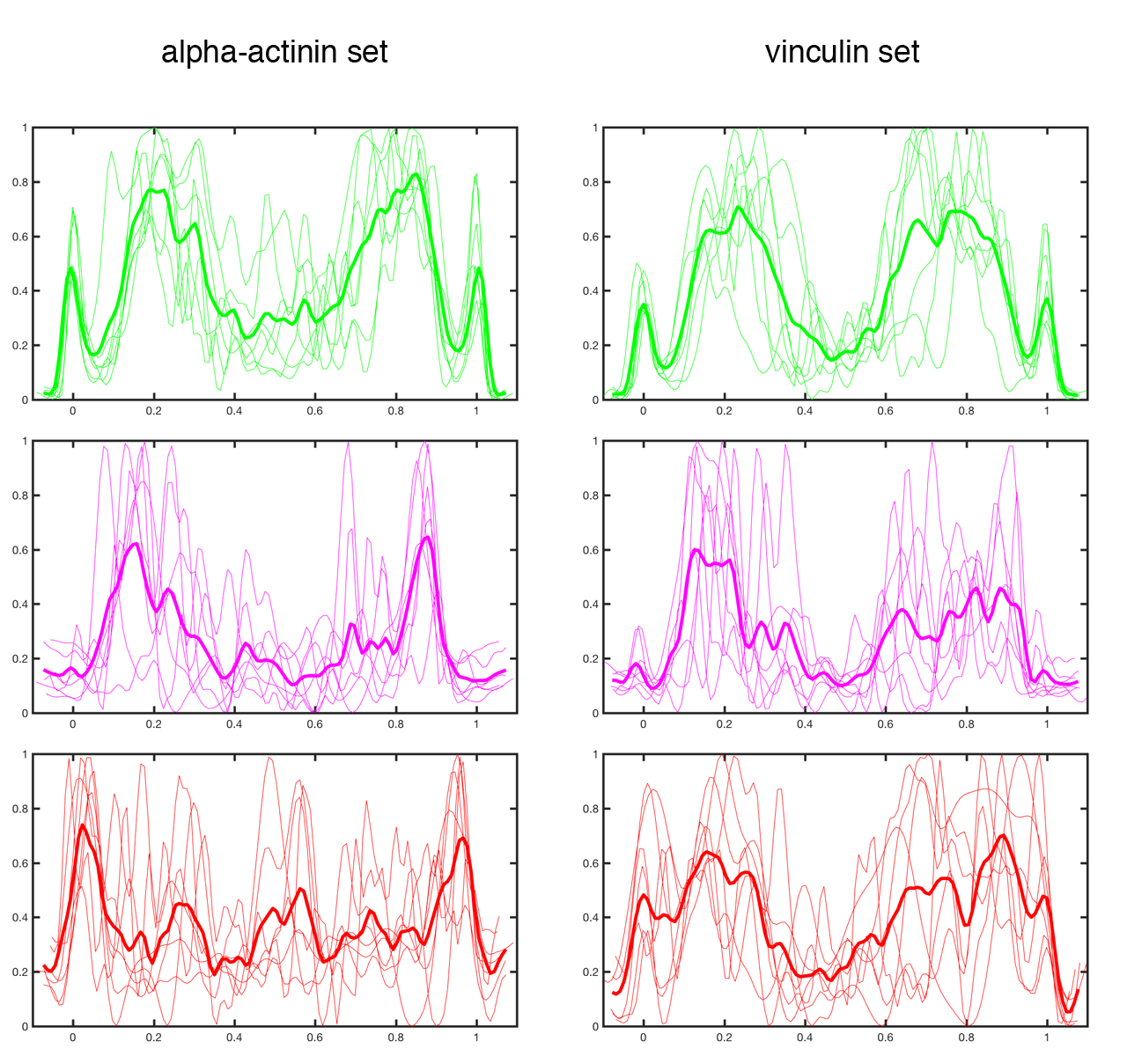

Supplement: S3 Fig — Line scan fluorescence intensity profiles of individual platelet cross-sections displaying distribution of actin (green), Pdlim7 (magenta) in conjunction with α-actinin (red) (left panels), and vinculin (red) (right panels). Intensities were analyzed on a line segment (width 380 nm) passing close to the center of each cell. Length of the line scan was normalized to 0 to 1 distance (x-axis) (left panel 3.47–6.04 μm; right panel 3.22–5.66 μm, n = 7 platelets, each set), using the cortical actin ring as start and end point. Along the diameter of the cell, fluorescence intensities were quantified and normalized to 0 to 1 (y-axis), representing minimum and maximum fluorescence of the individual proteins for each platelet scanned. The individual scans of a given protein were averaged over 7 platelets for each set (bold line). The mean normalized fluorescence profiles of each protein set were used for line scan panels of Fig 6I and 6J, respectively. (TIF) [file pone.0164042.s003.tif]

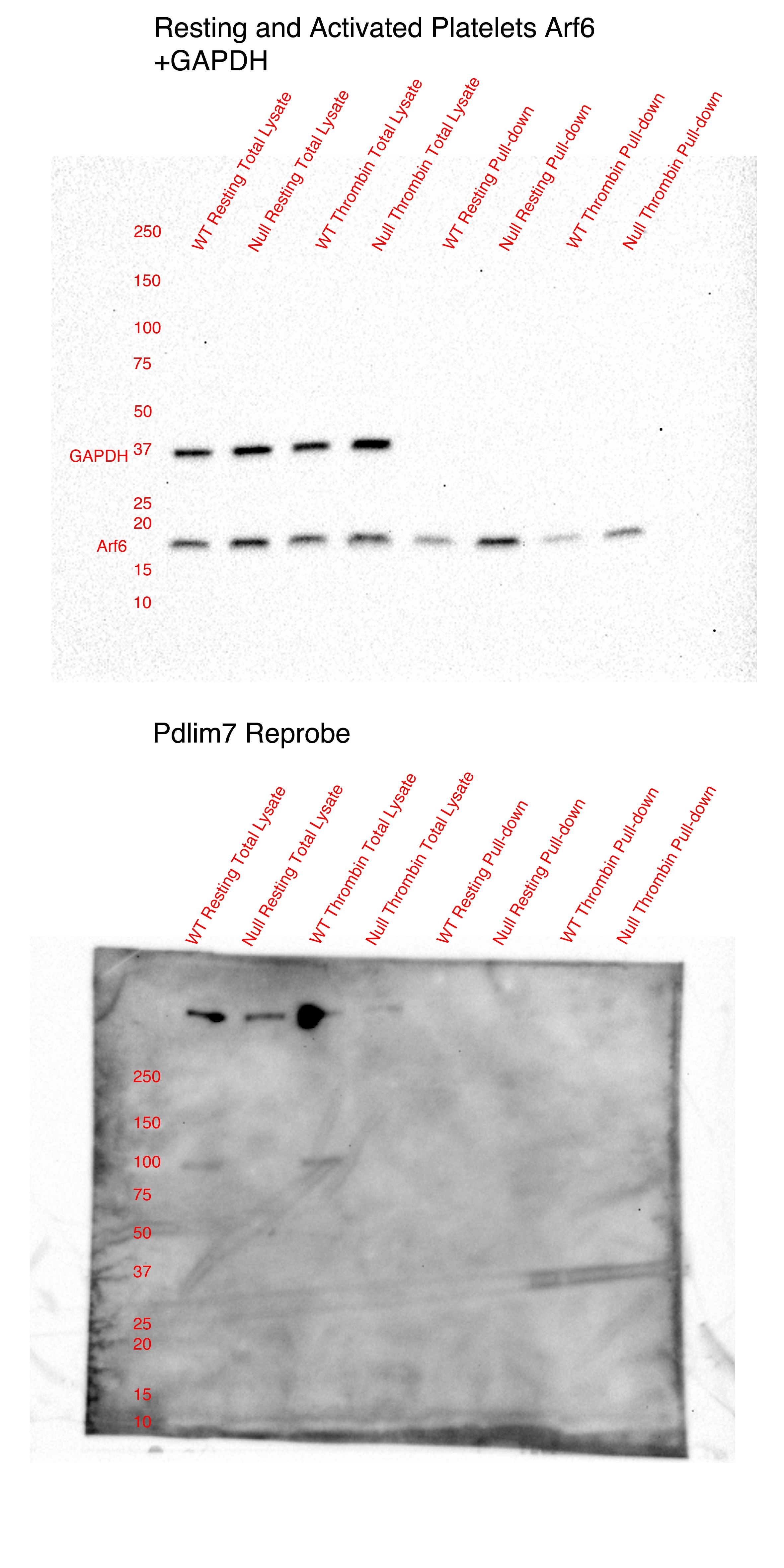

Supplement: S4 Fig — Top image shows Western blot processed with primary antibodies against Arf6 and GAPDH, and secondary goat anti-rabbit HRP-coupled antibody for visualization of proteins. Bottom image shows the same membrane stripped and re-probed with primary antibody against Pdlim7 in combination with goat anti-rabbit HRP-coupled secondary antibody as before. Protein lysates from WT and Pdlim7-/- mice were analyzed, in addition to samples from pull-downs that display the active, GTP-bound form of Arf6. Labels along the top of the images indicate sample loading. Images were obtained using a BioRad ChemiDoc MP system. (TIF) [file pone.0164042.s004.tif]
